# Effects of Walnut Consumption on Mood in Young Adults—A Randomized Controlled Trial

**DOI:** 10.3390/nu8110668

**Published:** 2016-10-25

**Authors:** Peter Pribis

**Affiliations:** Nutrition Program, Department of Individual, Family & Community Education, College of Education, University of New Mexico, Hokona Hall 157 MSC05 3040, Albuquerque, NM 87131-0001, USA; pribis@unm.edu; Tel.: +1-505-277-9612

**Keywords:** randomized controlled trial, RCT, walnuts, males, mood

## Abstract

Walnuts contain a number of potentially neuroprotective compounds like vitamin E, folate, melatonin, several antioxidative polyphenols and significant amounts of ω-3 fatty acids. The present study sought to determine the effect of walnuts on mood in healthy volunteers. Sixty-four college students were randomly assigned to two treatment sequences in a crossover fashion: walnut–placebo or placebo–walnut. At baseline mood was assessed using Profiles of Mood States (POMS). Data was collected again after eight weeks of intervention. After six-weeks of washout, the intervention groups followed the diets in reverse order. Data was collected once more at the end of the eight-week intervention period. No significant changes in mood were observed in the analyses with both genders combined and in females. However, we have observed a significant medium effect size improvement in the Total Mood Disturbance score (−27.49%, *p* = 0.043, Cohen’s d = 0.708) in males. In non-depressed healthy young males, walnuts seem to have the ability to improve mood.

## 1. Introduction

Numerous epidemiological and clinical trials have suggested that regular nut consumption has a beneficial effect on many health outcomes such as coronary heart disease, hypertension, diabetes, gallstones, weight gain over time, obesity, visceral obesity, metabolic syndrome, cancer and total mortality [1,2,3,4,5,6,7]. A number of studies have suggested significant protective effect of nuts against depression, mild cognitive disorders and Alzheimer’s disease [1]. Nuts, alone or as part of healthy dietary patterns, may exert beneficial effects due to their richness in antioxidants, including vitamins, polyphenols and unsaturated fatty acids, that may be protective against the development of cognitive decline and depression [8,9]. Walnuts contain several neuroprotective compounds like vitamin E, folate, melatonin and several polyphenols [10,11] (Table 1). Furthermore, walnuts also contain ω-3 α-linolenic acid (ALA; 18:3*n*-3) that can directly interact with the physiology of the brain. ALA is the precursor of Eicosapentaenoic acid (EPA; 20:5*n*-3) and Docosahexaenoic acid (DHA; 22:6*n*-3) [12]. DHA is an important ω-3 fatty acid (FA) responsible, among others, for membrane stability, neuroplasticity, speed of signal transduction, and modulation of serotonin and dopamine concentrations [13]. There is growing evidence that the synergy and interaction of all the nutrients and other bioactive components in nuts have beneficial effect on brain and cognition [12]. Studies regarding the effects of nuts consumption on cognitive functions are scarce. Most of the research on the relationship between nut consumption and cognitive functions has been done using animals or pathological populations [14,15]. There are presently no studies that investigated whether nut intake could be differently associated with changes in mood in men and women. Our study is also the only intervention study so far performed on healthy subjects. Thus, we have designed our study to evaluate the effects of walnut consumption on mood in a population of healthy young volunteers. Results suggest that regular walnut consumption in young males could improve mood.

## 2. Materials and Methods

The methodology of the study is described in great detail elsewhere [16], therefore here only briefly.

### 2.1. Subjects

Following an advertisement campaign on the campus of Andrews University we recruited 273 subjects. Subjects were disqualified if they were not students, not between the ages of 18 to 25 years, indicated that they would not be present on the campus the whole school year 2009/2010, did not complete a comprehensive Lifestyle survey and a short Food Frequency Questionnaire (FFQ), had known food allergies or did not participate in a comprehensive screening interview. The detailed participants’ flow diagram has been published elsewhere [16]. Eventually, 64 subjects were selected for the study.

### 2.2. Study Design

We used a double-blind, randomized, placebo-controlled, cross-over design. The 64 subjects were randomly assigned to one of the two study groups: walnuts–placebo and placebo–walnuts. Participants in the experimental group consumed the test meal (banana bread with walnuts) for the first eight weeks followed by six weeks of washout and another eight weeks of placebo diet (banana bread without walnuts). The other experimental group followed the diets in reverse order. The participants were asked to maintain their usual activities and other lifestyle habits throughout the duration of the study. The study personnel performing measurements and analyses were blinded to the subjects’ diet sequence. The subjects were offered $200 honorarium for their study participation.

The Institutional Review Board of Andrews University approved the study (IRB Protocol # 08-078).

### 2.3. Dietary Intervention

Participants were asked to consume three slices of banana bread every day for sixteen weeks (eight weeks banana bread with walnuts and eight weeks banana bread without walnuts). The two banana breads (walnut and placebo) were baked at the university’s kitchen and were very similar in appearance and taste. For the banana bread with walnuts, fresh, finely ground walnuts were mixed into the dough and baked at 177 °C (350 °F) for 1.5 h. We used English Walnuts (*Juglans regia* L.) for the dietary supplementation. Three slices of banana bread with walnuts contained two servings (½ cup or 60 g) of ground walnuts. Composition of the walnut and placebo banana breads is in Table 2. The test meal consumption was verified using plasma concentrations of ALA and Linoleic acid. Mean plasma concentrations of ALA and LA increased 68% and 11% when participants were consuming the banana bread with walnuts in comparison to placebo [16].

### 2.4. Mood Testing

The mood of the students was tested at baseline and at the end of each of the two eight-week treatment periods. The Profile of Mood States (POMS) questionnaire was used to estimate the intensity of mood disturbance in the participants. The POMS is one of the most widely used and accepted mood scales in studies of cognition [17,18]. The long form version of POMS consists of 65 adjectives. Participants were asked to read each item and respond to a 5-point Likert scale ranging from 1 (*Not at all*) to 5 (*Extremely*) based on how they felt at the time of questionnaire completion. The POMS covers six mood domains: Tension-Anxiety, Depression-Dejection, Anger-Hostility, Vigor-Activity, Fatigue-Inertia, and Confusion-Bewilderment. Summing the five negative domain scores and subtracting the vigor score computes the Total Mood Disturbance score (TMD). Higher scores indicate a greater degree of mood disturbance. Participants completed the questionnaire in a group setting under the guidance of a research assistant who explained the test to the participants and was available to answer questions during the administration. It took the participants approximately 30 to 45 min to complete the test.

The pre-specified primary outcome measure of our study was the TMD score. The secondary outcome measures were the six mood domains.

### 2.5. Statistical Analysis

The sample size determination was based on 5% significance, 95% power and measurable medium to large effect size of 0.5 to 0.7 SD. Allowing for a 30% dropout rate, the total sample size required for the study was 64 individuals.

Descriptive values are expressed as means ± SD or percentages. The analyses were carried out for both sexes combined and sex-specific. For the cross-over analysis, the SAS statistical software (version 9, SAS institute, Cary, NC, USA) used the mixed models procedure (PROC MIXED). Sequence, treatment and period were fixed effects and the subject was treated as a random effect. We tested for possible interactions between the dietary treatment and the sequence of the testing period. When differences reached significance, effect sizes (Cohen’s d) were calculated for determination of the clinical relevance of the observed effects. A d between 0.20 and 0.49 was considered to represent a small effect, a d between 0.50 and 0.79 was considered a medium effect, and a d greater than or equal to 0.80 was considered a large effect [19]. Two-sided *p* values less than or equal to 0.05 were considered statistically significant.

## 3. Results

### 3.1. Description of the Study Population

The selected baseline characteristics of the study population by study groups are presented in Table 3. All the participants were between the ages 18 to 25; the majority of students had normal weight. Majority of the study participants were Caucasian, followed by African American, Hispanic, Asian and participants with mixed ethnicity. The majority of the students (90%) were undergraduates. There were no statistical differences between the study groups.

During the study 17 (27%) of the participants dropped out. Six withdrew during the first week because of lack of interest; two transferred to a different college during the study and one was dropped because of no-compliance. Eight were excluded because they contracted the H_1_N_1_ flu and were not able to consume the test meal for several weeks. The dropout was similar in both study groups.

Consumption of three slices of banana bread daily changed the food intake of the participants. Three slices of banana bread with walnuts represented 4354 kJ (1040 kcal), three slices of banana bread without walnuts 3245 kJ (775 kcal). Using a FFQ we observed that during the dietary intervention, males consumed less milk, cereals, pasta and bread but drank more soy or rice milk; females consumed less milk, cheese, dried fruits, and cereals. During the first eight weeks the average weight increase for the walnut group was 1.3 kg (SD 1.3) and 0.9 kg (SD 1.0) for the placebo group (*p* < 0.001 for both diets). During the second eight weeks weight increased 0.4 kg (SD 1.4) for the walnut group and 0.5 kg (SD 2.2) for the placebo group (*p* = 0.195 for walnuts; *p* = 0.279 for placebo).

### 3.2. POMS Results

All calculations for POMS are based on raw scores. Table 4 presents the mean scores and treatment effects for both sexes combined. No significant changes were observed for any of the domains and the TMD score. In males (Table 5) we observed a significant medium effect size improvement of the TMD score when on the walnut diet (−27.49%, *p* = 0.043, Cohen’s d = 0.708). No significant changes in any of the POMS scores were observed in females (Table 6).

## 4. Discussion

In this double-blind, randomized, placebo controlled, cross-over trial supplementation with walnuts for eight weeks we did not observe any improvement in mood in the combined analysis and in females. However, in young healthy males we have observed a significant, medium effect size improvement of the TMD score.

There are very few studies regarding nut consumption and depression. Pooled meta-analysis, synthesizing results of nine studies (one longitudinal cohort, one case-control and seven cross-sectional) examining the association between adherence to a Mediterranean diet (MD) and depression, showed a 32% reduction in the risk of depression (RR = 0.68, 95% CI 0.54–0.86) in individuals with high dietary adherence to the MD. The protective effect of high adherence to the MD was independent of age, whereas moderate adherence lost its protective properties in older age [20]. MD is characterized by high intake of vegetables, fruits, cereals, pulses, nuts and seeds; moderate consumption of dairy products, fish, poultry and eggs; generous use of olive oil and moderate intake of wine with meals [21]. In the PREDIMED trial, a nutritional intervention with the MD supplemented with nuts showed a 40% lower risk of depression in participants with type 2 diabetes (RR = 0.59, 95% CI 0.36–0.98). However, the inverse association with depression was not significant (RR = 0.78, 95% CI 0.55–1.10) in the whole cohort [22]. However, the evidence for the protective effect of nuts from these studies is only indirect in the setting of a healthy MD.

There are several nutrients in walnuts that could be responsible for the reduced risk of depression and improved mood. Walnuts are different from other tree nuts because of their greater antioxidant capacity, polyphenols and ALA content. Walnuts are an excellent source of ω-3 FA; 28 g of walnuts (recommended daily dose) contains 2.6 g of ALA. ALA found in walnuts is associated with improved endothelial function and inflammation in animal models [23]. Polyunsaturated Fatty Acids (PUFA) and Monounsaturated Fatty Acids (MUFA) content may contribute to the improvement in arterial stiffness [24]. Still, PUFA and specifically ALA can also directly influence brain physiology including structural changes in brain areas associated with affective experiences [25]. ALA is the precursor for DHA that can modulate serotonin and dopamine concentration known to influence mood and sleep [13]. The present, available evidence provides some support that ω-3 PUFA intake is associated with reduced depressive symptoms particularly in females [26,27]. There are several factors that could contribute to sex-based differences in ω-3 PUFA intake and mood. DHA levels are higher in women than men due to sex hormones; estrogen increases DHA, while testosterone decreases DHA levels [28]. Conversion of ALA to DHA is higher in women than men [29]. But, some studies found no sex-based differences [30], some found opposite sex effect [31]. There have also been few studies that examined the effect of ω-3 PUFA supplementation on mood in young healthy populations. Fontani et al. reported that ω-3 PUFA supplementation (2.8 g/day) using POMS increased feelings of vigor and reduced feelings of anger, anxiety, fear, depression and confusion [32]. But, Antypa et al. found that 2.3 g/day of ω-3 PUFA supplementation only reduced feelings of fatigue in POMS but not depression in POMS or Beck Depression Inventory [33]. Young reported that ω-3 PUFA algae supplementation for eight weeks resulted in increased overall mood disturbance as measured by POMS and Depression Anxiety Stress Scale [34]. Vitamin E and polyphenols can modify inflammatory mediators [35,36]. This vascular and anti-inflammatory benefit alone could contribute to improving cognitive functions including mood. Walnuts contain relatively high levels of food-based melatonin (3.5 ng/g), which can act as powerful antioxidant, and regulator of biological rhythmicity and sleep [37]. Sufficient sleep has been associated with better mood [38]. Lastly, walnuts are also rich source of folate. Folate sufficiency is associated with less cognitive impairment and depression [39].

However, all these results do have only limited applicability because our study was a whole food study [40]. We have measured the effects of the consumption of the whole walnut on mood with all the possible synergy and interactions between all the nutrients and compound found there.

This is the first intervention study in humans exclusively measuring the effect of walnut consumption on mood. Both males and females in our study scored around the 50th percentile at baseline, indicating that our population was not depressed. Walnut supplementation was able to improve mood only in males. It is not clear why we didn’t observe any mood changes in females.

The TMD score is computed by summing up the five negative domains and subtracting the vigor score. Male participants, when on the walnut diet, experienced non-significant improvements in: tension (−18.61%), depression (−26.52%), anger (−31.15%), vigor (13.42%) and confusion (−6.35%), with no changes in fatigue. When added up, this led to a significant medium size improvement in the TMD score (−27.49%, *p* = 0.043, Cohen’s d = 0.708). Our results are intriguing because small improvements attributable to walnut consumption in healthy, cognitively intact young adults could translate into important outcomes in aging populations [8]. This sex specific influence of walnuts on mood, we have observed, is far from understood and warrants more research.

There are several limitations to our study. The duration of the study may have been too short. We experienced unusually high drop-out rates because of the H_1_N_1_ flu epidemic. This attrition rate reduced the level of statistical power available for our analysis. However, the bias that would result from withdrawal of participants with different moods is likely to be negligible. Participant expectation of treatment effects could have altered the results.

Clinical trials often require multiple outcomes and multiple hypotheses to be tested. Such testing involves comparing treatments using multiple outcome measures (MOMs) with univariate statistical methods [41]. Some researchers argue that if MOMs are tested, the *p*-values should be adjusted upward because of the multiple testing problem [42]. Applying this conservative approach, results for the TMD score for males would be insignificant because the reference *p* value would be 0.05/2 = 0.025 (α divided by the two genders). However, objection to *p*-values adjustments is that the significance of each test will be interpreted according to how many outcome measures are considered in the family-wise hypothesis and additionally while reducing type I error we increase the chance of making type II error. Therefore, we have used the following recommended strategy to reach reasonable conclusions [41]: our study was a randomized double blind controlled trial that followed the CONSORT guidelines; the effect size on the TMD score for males was medium (Cohen’s d = 0.708); the focus of the publication are only the primary outcomes—the TMD scores. It is important that further research be done to replicate our results.

## 5. Conclusions

In conclusion, in this randomized, double-blind, placebo-controlled feeding trial, supplementation with walnuts was able to improve mood in healthy, non-depressed males. No effect on mood was observed in females.

## Figures and Tables

**Table 1:** Selected nutrients in Walnuts.

Nutrients in 14 Halves (28 g)	
**Minerals** (mg)	
Calcium	28
Magnesium	45
Potassium	125
**Vitamins** (μg)	
Thiamin	97
Vitamin B6	152
Folate	28
Lutein	3
α-tocopherol	200
β-tocopherol	40
γ-tocopherol	5910
δ-tocopherol	540
**Lipids** (g)	
ALA; 18:3*n*-3	2.6
LA; 18: 2*n*-6	10.8
**Phenols** (mg GAE)	
Total phenols	436
**Other** (ng)	
Melatonin	98

Data are for raw English Walnut; All data are from references [10,11]; ALA, α-linolenic acid; LA, linoleic acid; GAE, gallic acid equivalents.

**Table 2:** Composition of the banana bread.

Per 100 g	Walnuts	Placebo
Energy (kJ/kcal)	1612/385	1202/287
Carbohydrate (g)	40.9	48.9
Protein (g)	7.0	5.2
Fat (g)	21.5	7.9
Saturated Fatty Acids (g)	2.5	1.5
Monounsaturated Fatty Acids (g)	3.8	1.9
Polyunsaturated Fatty Acids (g)	14.1	4.0
α—Linolenic acid (18:3*n*-3) (g)	2.5	0.2
Linoleic acid (18:2*n*-6) (g)	12.2	4.0

Values obtained by chemical analysis (Covance laboratories, Madison, Wisconsin).

**Table 3:** Selected baseline characteristics of the participants by study groups.

Group	Walnut–Placebo (*n = 32*)		Placebo–Walnut (*n = 32*)		*p*
	Mean	SD	Mean	SD	
Age (years)	20.6	2.0	20.7	2.1	0.872 *
BMI (kg/m^2^)	22.6	3.3	23.2	3.5	0.471 *
	*n*	*%*	*n*	*%*	
Ethnicity (*n*, %)					0.255 ^†^
Caucasian	18	56.3	11	35.5	
African American	5	15.6	7	22.6	
Other	9	28.1	13	41.9	
Class standing (*n*, %)					0.977 ^†^
Freshman	8	25.0	7	22.6	
Sophomore	7	21.9	9	29.0	
Junior	8	25.0	7	22.6	
Senior	6	18.8	5	16.1	
Graduate	3	9.4	3	9.7	

* Independent means *t*-test; ^†^ Chi-square test; SD, Standard deviation.

**Table 4:** Mean scores and changes in Profiles of Mood States (POMS) for both sexes on walnut and placebo diet (*n* = 49).

	Treatment Means		Treatment Effect	
	Walnuts	Placebo	Walnuts–Placebo	
**Domains/TMD**	**Mean (95% CI)**	**Mean (95% CI)**	**% Change (95% CI)**	***p*-Value**
Tension-Anxiety	11.2 (9.3–13.2)	11.9 (9.9–13.8)	−5.2 (−20.8–10.4)	0.507
Depression-Dejection	9.7 (6.7–12.7)	12.1 (9.1–15.2)	−20.0 (−44.2–4.2)	0.103
Anger-Hostility	6.5 (4.7–8.3)	7.5 (5.7–9.3)	−13.3 (−36.8–10.2)	0.261
Vigor-Activity	13.8 (12.2–15.4)	13.1 (11.5–14.7)	5.3 (−7.5–18.0)	0.408
Fatigue-Inertia	10.2 (8.7–11.7)	10.1 (8.6–11.6)	1.1 (−14.3–16.6)	0.882
Confusion-Bewilderment	9.5 (8.2–10.8)	9.5 (8.2–10.8)	0.0 (−12.6–12.1)	0.969
**Total Mood Disturbance**	33.5 (24.7–42.3)	37.9 (29.1–46.6)	−11.6 (−32.4–9.2)	0.267

Means ± 95% Confidence intervals; TMD, Total Mood Disturbance; CI Confidence intervals.

**Table 5:** Mean scores and changes in POMS for males on walnut and placebo diet (*n* = 20).

	Treatment Means		Treatment Effect	
	Walnuts	Placebo	Walnuts–Placebo	
**Domains/TMD**	**Mean (95% CI)**	**Mean (95% CI)**	**% Change (95% CI)**	***p*-Value**
Tension-Anxiety	9.1 (6.2–12.0)	11.2 (8.35–14.0)	−18.6 (−42.4–5.2)	0.117
Depression-Dejection	9.4 (4.5–14.4)	12.9 (8.01–17.7)	−26.5 (−61.9–8.9)	0.132
Anger-Hostility	6.5 (3.4–9.6)	9.4 (6.4–12.5)	−31.2 (−54.9–−7.4)	0.013
Vigor-Activity	15.6 (12.9–18.2)	13.8 (11.18–16.3)	13.4 (−4.7–31.5)	0.136
Fatigue-Inertia	10.0 (7.3–12.8)	10.3 (7.57–13.1)	−2.8 (−21.2–15.6)	0.750
Confusion-Bewilderment	9.3 (6.9–11.7)	9.9 (7.6–12.3)	−6.4 (−23.0–10.3)	0.432
**Total Mood Disturbance**	28.9 (13.3–44.7)	39.9 (24.4–55.6)	−27.5 (−54.0–−1.00)	0.043

Means ± 95% Confidence intervals; TMD, Total Mood Disturbance; CI Confidence intervals.

**Table 6:** Mean scores and changes in POMS for females on walnut and placebo diet (*n* = 29).

	Treatment Means		Treatment Effect	
	Walnuts	Placebo	Walnuts–Placebo	
**Domains/TMD**	**Mean (95% CI)**	**Mean (95% CI)**	**% Change (95% CI)**	***p*-Value**
Tension-Anxiety	12.6 (9.9–15.2)	12.1 (9.5–14.8)	3.6 (−18.3–25.6)	0.735
Depression-Dejection	9.7 (5.7–13.8)	11.3 (7.1–15.4)	−13.6 (−49.8–22.6)	0.444
Anger-Hostility	6.4 (4.0–8.7)	6.0 (3.6–8.4)	6.5 (−36.7–49.7)	0.758
Vigor-Activity	12.6 (10.5–14.7)	12.6 (10.4–14.7)	0.3 (−18.5–19.1)	0.974
Fatigue-Inertia	10.1 (8.3–12.0)	9.9 (8.0–11.8)	2.2 (−21.7–26.0)	0.853
Confusion-Bewilderment	9.6 (8.0–11.2)	9.1 (7.5–10.8)	5.1 (−13.9–24.0)	0.587
**Total Mood Disturbance**	35.7 (24.8–46.6)	35.6 (24.5–46.6)	0.4 (−32.3–33.0)	0.982

Means ± 95% Confidence intervals; TMD, Total Mood Disturbance; CI Confidence intervals.
